# Serum α-Klotho Protein Can Be an Independent Predictive Marker of Oxidative Stress (OS) and Declining Glomerular Function Rate in Chronic Kidney Disease (CKD) Patients

**DOI:** 10.7759/cureus.25759

**Published:** 2022-06-08

**Authors:** Sudeep Jena, Pratikhya Sarangi, Upendra K Das, Andrew A Lamare, Roma Rattan

**Affiliations:** 1 Biochemistry, SCB Medical College and Hospital, Cuttack, IND; 2 Biochemistry, Pandit Raghunath Murmu Medical College, Baripada, IND; 3 Biochemistry, Government Medical College (GMC) Sundergarh, Cuttack, IND

**Keywords:** reactive oxygen species, inflammatory marker, glomerular function rate, chronic kidney disease, α-klotho

## Abstract

Introduction

Chronic kidney disease (CKD) has been recognized as a global health problem. Progression of CKD to advanced stages is associated with a significant increase in the generation of reactive oxygen species (ROS). An antiaging protein, α-Klotho, is found expressed in the distal convoluted tubules of the kidney where, predominantly, it works to increase calcium absorption and potassium excretion in distal tubule via N-linked glycans. The association of serum α-Klotho with oxidative stress, inflammation, and fibrosis, as seen in CKD, highlights its importance for studying disease prognosis with declining glomerular function rate (GFR).

Material and methods

This was a case-control study consisting of 90 subjects. Fifty diagnosed cases of CKD attending the department of nephrology, SCB Medical College, Cuttack, Odisha, were included, and 40 age and sex-matched healthy volunteers were taken as control. Serum α-Klotho levels were measured using enzyme-linked immunosorbent assay kits. Oxidative stress by estimating the total oxidant load by ferrous oxidation-xylenol orange version 2 (FOX2) method and the total antioxidant capacity of serum by the ferric reducing ability of plasma (FRAP) method. Estimation of the estimated glomerular filtration rate (eGFR) was done using the Cockcroft and Gault equation.

Results

Serum α-Klotho (ng/ml) was found to be 2.59±0.98 in cases as compared to 0.24±0.09 in controls (p< 0.01). The serum total oxidant load (ng/ml) was 1.96±1.01 and 0.05±0.02 in cases and controls, respectively. Serum total antioxidant capacity (µM) was measured as 281.80±78.0 in cases and 862.82±51.86 in controls. (p< 0.01). Serum Klotho has a negative correlation with eGFR in CKD patients (r = -0.065; p = 0.648).

Conclusion

The serum α-Klotho level was significantly higher in CKD patients than in healthy volunteers. Both serum α-Klotho and oxidative stress were negatively correlated with eGFR in CKD patients. Serum α-Klotho can be a suitable biomarker in CKD patients with declining GFR.

## Introduction

Chronic kidney disease (CKD) can be defined as any structural and/or functional damage to the kidneys or any decline in the glomerular filtration rate (GFR) below 60 mL/min/1.73 m^2^, for at least three months or more, resulting from any cause [1]. Most chronic diseases like diabetes mellitus and hypertension, as well as some primary renal disorders like glomerulonephritis eventually develop CKD as their long-term complication [2]. The global estimated prevalence of CKD is 13.4% (11.7-15.1%), and patients with end-stage kidney disease (ESKD) needing renal replacement therapy are estimated at between 4.902 and 7.083 million [3]. Although data are scarce for developing countries, it is estimated that by 2030, 70% of patients with end-stage renal disease (ESRD, stage 5 CKD), will be in developing countries, which increases the burden on the budgetary capabilities of health care systems [2].

α-Klotho protein is a transmembrane protein encoded by the anti-aging gene KL. It was discovered by Kuro-O in 1997 and is predominantly expressed in the distal convoluted tubules of the kidney but is also expressed in the brain choroid plexus, pituitary gland, pancreas, and reproductive organs [4]. α-Klotho exists in two forms, i.e. one membrane-bound form and a soluble form. The mechanism of action of α-Klotho protein is to increase calcium ion absorption and potassium ion excretion in distal convoluted tubules of the kidneys via receptors like N-linked glycans transient receptor potential cation channel subfamily V member 5 (TRPV5) and renal outer medullary potassium channel (ROMK1).

In CKD, there is evidence of early reduction of renal Klotho mRNA expression, which affects both transmembrane Klotho and soluble Klotho. This is considered to be responsible for the development of kidney tubular cell resistance to fibroblast growth factor (FGF23). FGF23 regulates both the renal handling of phosphate and renal synthesis of calcitriol [5]. Soluble Klotho may have anti-inflammatory, anti-apoptotic effects [5].

CKD progression is associated with increased oxidative stress (O.S). O.S is an imbalance between free radicals and antioxidants. Impaired mitochondrial respiratory system in CKD patients is suggested both as the consequence and the cause of enhanced oxidative stress, which may explain a subnormal energy metabolism in this population. The progression of CKD to advanced stages is associated with a significant increase in the generation of ROS [6]. Klotho protein has been studied to play a significant role in the insulin pathway by inhibiting insulin-like growth factor (IGF) signaling [7]. It increases resistance to oxidative stress at both the cellular and organismal level in mammals. It activates FOXO (forkhead box O transcription factor) that is negatively regulated by insulin/IGF1 signaling, thereby inducing the expression of manganese superoxide dismutase. This, in turn, facilitates the removal of reactive oxygen species and confers oxidative stress resistance. Thus, Klotho-induced inhibition of insulin/IGF-1 signaling is associated with increased resistance to oxidative stress [7].

## Materials and methods

This was a case-control study comprising 90 samples. Fifty diagnosed cases of chronic kidney disease of any stage attending the department of nephrology, S.C.B. Medical College, Cuttack, Odisha, were included, and 40 age and sex-matched normal healthy volunteers were taken as control. The study was conducted at the Molecular Genomic Laboratory, Department of Biochemistry, in collaboration with the Department of Nephrology, S.C.B. Medical College, Cuttack, Odisha. Informed consent from all enrolled subjects was obtained for participation in this study. We have included in our study all clinically diagnosed cases of CKD according to the criteria given in Table 1. The exclusion criteria with reference to Table 1 are patients suffering from another endocrine autoimmune disorder.

**Table 1:** Inclusion and exclusion criteria for the study population CKD: chronic kidney disease

INCLUSION CRITERIA	EXCLUSION CRITERIA
1) Clinically diagnosed cases of chronic kidney disease with diabetes mellitus	1) Patients with any endocrine disorder other than diabetes
2) Clinically diagnosed cases of chronic kidney disease with hypertension	2) Chronic smoker
3) Clinically diagnosed cases of idiopathic chronic kidney disease	3) Chronic alcoholic
4) CKD patients on dialysis	4) Presence of any autoimmune disorder

Ethical statement

This study has been approved by the Institutional Ethics Committee (Registration No. ECR/84/Inst/OR/2013 issued under Rule 122DD of the Drugs and Cosmetics Rules 1945) under the IEC/IRB No- 805/11.3.2019.

Key anthropometric measurements like weight, height, BMI, blood pressure along with pulse rate were noted. Serum samples were collected and FBS, serum urea, creatinine, total protein values, and electrolytes were estimated in the autoanalyzer of both the cohorts by using kits adapted to the autoanalyzer. Serum α-Klotho was estimated by an enzyme-linked immunosorbent assay (ELISA) kit in an automated ELISA system Evolis Twin Plus (Bio-Rad Laboratories, Hercules, California). Oxidative stress was done by estimating total oxidant load by the ferrous oxidation-xylenol orange version 2 (FOX2) method and total antioxidant capacity of serum by the ferric reducing antioxidant power (FRAP) method both by ELISA. Estimation of the estimated glomerular filtration rate (eGFR) was done using the Cockcroft and Gault equation [8].

CCr= {((l40-age) x weight)/(72xSCr)}x 0.85 (if female)

 CCr (Creatinine clearance) in mL/min; Age in years; Weight in kg, and SCr (serum creatinine) in mg/dL

Statistical analysis was done using SPSS 20 (IBM Inc, Chicago, Illinois). Results were expressed as mean ± standard deviation. Data were compared using the paired t-test and a p-value of < 0.05 was considered statistically significant. Pearson’s correlation coefficient (r) and linear regression analysis were done.

## Results

Table 2 shows the comparison of demographics, anthropometric parameters, and vitals among cases and controls. Age, gender ratio, weight, height, BMI, pulse, and BP were compared in both groups. All data were represented as mean ± standard deviation (SD). The data were compared by the unpaired student's t-test. A p-value of ≤ 0.05 was considered significant. We observed a significant difference (p < 0.01) in the gender ratio, weight, BMI, and systolic blood pressure (SBP) in CKD patients as compared to healthy volunteers.

**Table 2:** Comparison of demographics, anthropometric parameters, and vitals among study participants * p-value ≤ 0.05: significant CKD: chronic kidney disease

Parameters	Cases (n=50) (CKD patients)	Controls (n=40) (Healthy volunteers)	p-value	
Age (years)	48.72±10.98	42.60±11.08	0.011*	
Gender				
Male	38(76%)	15(37.5%)	< 0.01*	
Female	12(24%)	25(62.5%)		
Anthropometry				
Weight (kg)	65.98±10.07	60.22±7.20	0.003*	
Height (meters)	1.55±0.09	1.54±0.08	0.532	
Body mass index (kg/m^2^)	27.23±4.19	25.11±1.95	0.004*	
Vitals				
Pulse (per minute)	76 ± 5.48	74.75 ± 5.53	0.121	
Systolic blood pressure (mmHg)	120 ± 6.96	116.45 ± 5.62	0.002*	
Diastolic blood pressure (mmHg)	81 ± 4.31	79.70 ± 3.91	0.083	

Table 3 shows the comparison of biochemical parameters among cases and controls. Fasting blood sugar (FBS), serum urea, and creatinine were significantly higher (p <0.01) among CKD patients compared to the control group, whereas eGFR, serum sodium, potassium, calcium, and serum total protein were significantly higher (p < 0.01) among healthy volunteers when compared to CKD patients.

**Table 3:** Comparison of biochemical parameters among the study population * p-value ≤ 0.05: significant

Parameters	Cases (n=50)	Controls (n=40)	p-value
Serum fasting blood sugar (mg/dl)	132.28±44.76	96.62±8.61	< 0.01*
Renal Function Tests			
Serum Urea (mg/dl)	132.58±53.04	20.77±5.64	< 0.01*
Serum Creatinine (mg/dl)	10.31±4.25	0.82±0.19	< 0.01*
Estimated glomerular filtration rate (eGFR) (ml/min)	9.58±6.01	95.37±30.37	< 0.01*
Electrolytes			
Serum Sodium (mmol/l)	121.26±9.21	137.77±4.66	< 0.01*
Serum Potassium (mmol/l)	1.95±0.54	4.05±0.49	< 0.01*
Serum Calcium (mmol/l)	0.65±0.26	1.22±0.09	< 0.01*
Serum Total Protein (gm/dl)	3.48±0.47	5.95±0.81	< 0.01*

Table 4 depicts the comparison of serum α-Klotho, total oxidant load, and total antioxidant capacity among cases and controls. We observed a significantly higher p-value (< 0.01) for serum Klotho in CKD patients compared to the control group. The total oxidant load was significantly higher in CKD compared to controls, whereas the serum total antioxidant capacity value was significantly decreased in cases (p < 0.01) when compared to the control group.

**Table 4:** Comparison of special parameters among study participants * p-value ≤ 0.05: significant

Parameters	Cases (n=50)	Controls (n=40)	p-value
Serum α-Klotho (ng/ml)	2.59±0.98	0.24±0.09	< 0.01*
Serum total oxidant load (ng/ml)	1.96±1.01	0.05±0.02	< 0.01*
Serum total antioxidant capacity (µM)	281.80±78.0	862.82±51.86	< 0.01*

Table 5 shows the correlation of serum Klotho with anthropometric and biochemical parameters among cases and controls. Serum Klotho has a significant positive correlation with FBS (r = 0.352, p = 0.012) in CKD patients and with serum total protein (r = 0.380, p =0.016) in healthy volunteers. Whereas, it has a significant negative correlation with serum sodium in healthy volunteers. In CKD patients, renal function parameters and serum calcium have a positive correlation with no significant p-value.

**Table 5:** Correlation of serum Klotho with anthropometric and biochemical parameters among the study population * p-value ≤ 0.05: significant; r = Pearson’s correlation coefficient

Parameters	Cases (n=50)		Controls (n=40)	
	r value	p-value	r value	p-value
Body mass index (kg/m^2^)	0.109	0.737	-0.096	0.385
Pulse (per minute)	0.021	0.692	-0.198	0.611
Systolic blood pressure (mmHg)	0.117	0.452	-0.112	0.490
Diastolic blood pressure (mmHg)	0.122	0.998	-0.043	0.337
Serum fasting blood sugar (mg/dl)	0.352	0.012*	-0.210	0.388
Serum urea (mg/dl)	0.132	0.227	0.018	0.106
Serum creatinine (mg/dl)	0.106	0.744	-0.100	0.529
Serum sodium (mmol/l)	0.104	0.830	-0.234	0.033*
Serum potassium (mmol/l)	-0.021	0.632	0.317	0.265
Serum calcium (mmol/l)	0.076	0.224	0.162	0.441
Serum total protein (gm/dl)	0.188	0.190	0.380	0.016*

Table 6 shows the correlation of eGFR with serum Klotho, total oxidant load, and total antioxidant capacity among cases and controls. Serum Klotho has a negative correlation with eGFR in CKD patients with a significant p-value. Serum total antioxidant capacity has a significant negative correlation with eGFR in healthy volunteers.

**Table 6:** Correlation of eGFR with serum Klotho and oxidative stress among the study population * p-value ≤ 0.05: significant; r = Pearson’s correlation coefficient eGFR: estimated glomerular filtration rate

Parameters	Cases (n=50)		Controls (n=40)	
	r value	p-value	r value	p-value
Serum Klotho (ng/ml)	-0.065	0.648	0.064	0.741
Serum total oxidant load (ng/ml)	0.020	0.101	-0.081	0.175
Serum total antioxidant capacity (µM)	0.133	0.702	-0.051	0.004*

Table 7 shows the linear regression of serum Klotho, total oxidant load, and total antioxidant capacity with eGFR among cases and controls. The standardized β coefficient represented the slope and showed that for each unit decrease in eGFR, there was an increase of 0.070 units of serum Klotho in CKD patients and a decrease of 0.063 units in healthy volunteers. Similarly, for each unit decrease in eGFR, there was an increase in total oxidant load and a decrease in total antioxidant capacity in all study participants.

**Table 7:** Linear regression of serum Klotho and oxidative stress with eGFR among study participants eGFR: estimated glomerular filtration rate

Coefficient	Cases (n=50)	95% Confidence Interval (CI)	Controls (n=40)	95% Confidence Interval (CI)
Serum Klotho (ng/ml)	-0.070	-2.231 – 1.378	0.063	-0.920 – 1.264
Serum total oxidant load (ng/ml)	-0.098	-2.348 – 1.193	-0.020	-1.030 – 1.394
Serum total antioxidant capacity (µM)	0.044	-0.020 – 0.026	0.082	-0.040 – 0.046

**Figure 1:**
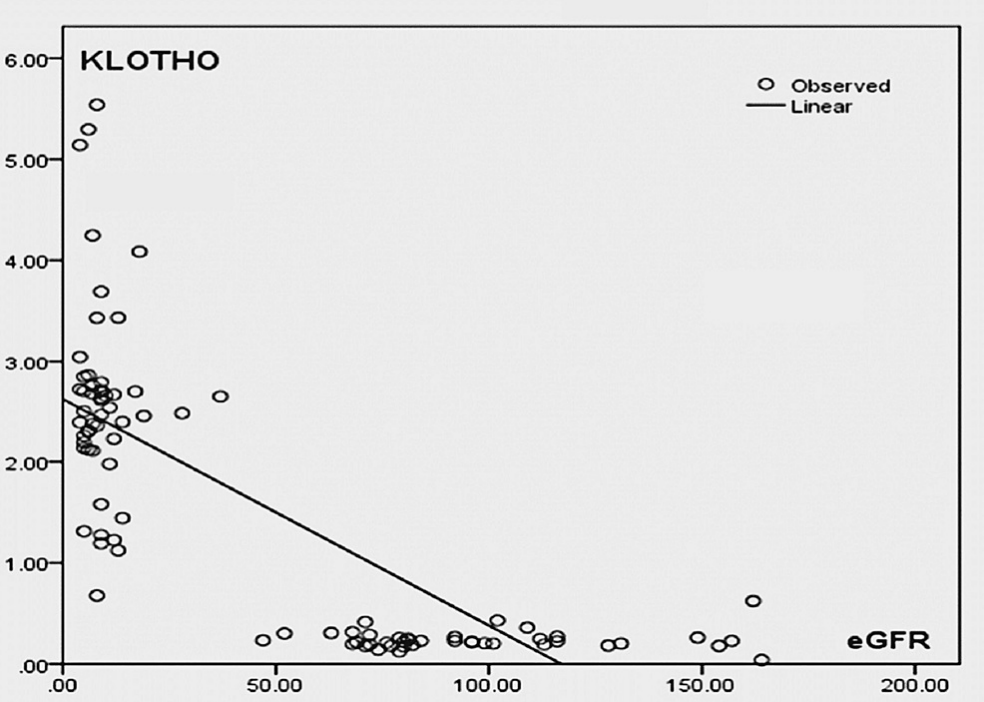
Linear regression analysis between eGFR (in ml/min) and serum Klotho (ng/ml) eGFR: estimated glomerular filtration rate

Figure 1 shows linear regression analysis between eGFR (in ml/min) and serum Klotho (in ng/ml). This scatter plot showed that with a decrease in eGFR, serum Klotho level increases in CKD patients. But the association between the variables was not statistically significant. (r = -0.065; p = 0.648).

**Figure 2:**
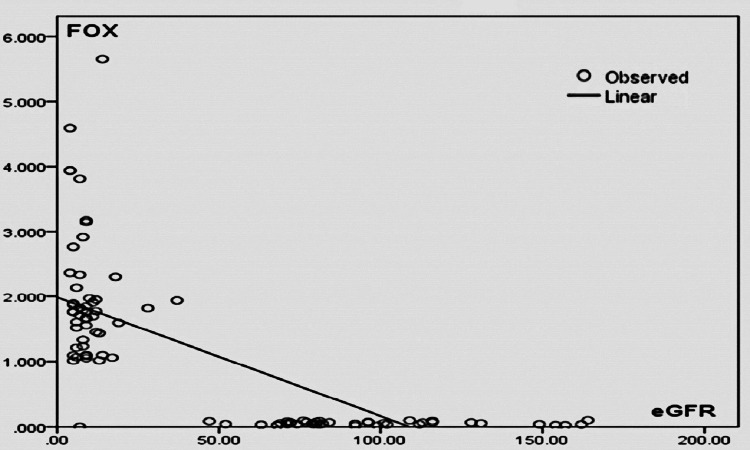
Linear regression analysis between eGFR (ml/min) and serum total oxidant load (FOX) (ng/ml) eGFR: estimated glomerular filtration rate; FOX: ferrous oxidation-xylenol orange

Figure 2 shows linear regression analysis between eGFR (in ml/min) and serum total oxidant load (FOX) (in ng/ml). This scatter plot showed that in CKD patients with a decrease in eGFR, there was an increase in oxidant load. But the association is not statistically significant (r = 0.020; p = 0.101).

**Figure 3:**
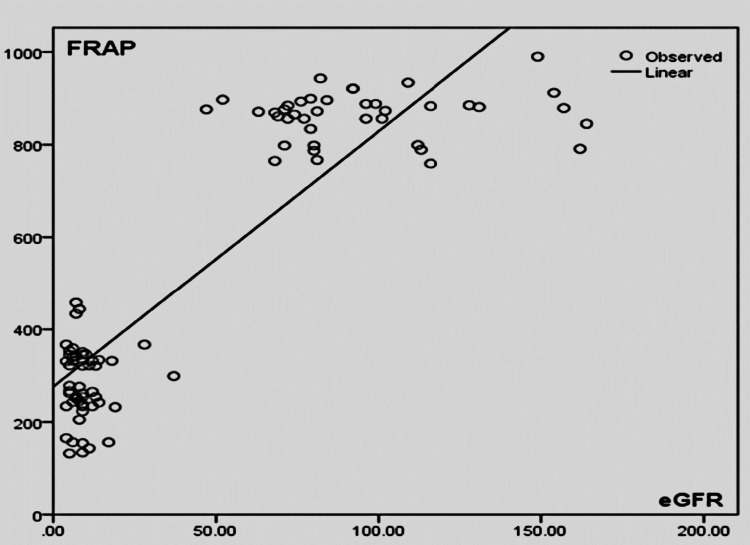
Linear regression analysis between eGFR (in ml/min) and serum total antioxidant capacity (FRAP) (in µM) eGFR: estimated glomerular filtration rate; FRAP: ferric reducing antioxidant power

Figure 3 shows linear regression analysis between eGFR (in ml/min) and serum total antioxidant capacity (FRAP) (in µM). In CKD patients, it was found that with a decrease in eGFR, there was a decrease in antioxidant capacity but it was statistically not significant (r = 0.133; p = 0.702).

## Discussion

Chronic renal disease is recognized as an important global health problem. Recent studies have implicated CKD to be the most important cause of end-stage renal disease (ESRD) and renal transplant. The global prevalence of CKD is 9.1% (697.5 million cases) [9]. In developing countries like India, chronic diseases like type 2 diabetes mellitus and hypertension are the major contributors to the development of CKD. In India, the prevalence of CKD patients stands at a rate of 800 per million population and the incidence of CKD patients progressing to ESRD is 150-200 per million population [10].

The pathophysiology of CKD involves a few broad mechanisms of renal damage such as (i) underlying genetic/systemic abnormalities in kidney development or integrity of glomerular membrane; (ii) damage due to the deposition of immune complex proteins or inflammatory products leading to glomerulonephritis or damage to renal tubules; (iii) long-standing mechanisms such as increased renal blood flow (hyperfiltration) and hypertrophy of the remaining viable nephrons [11]. In the kidneys, the nephrons are subjected to vasoactive hormones, growth factors, and cytokines, which grossly reduce their number. The nephrons adapt to these environments by hypertrophy and hyperfiltration, resulting in increased blood flow and pressure. This, in turn, leads to distortion of the glomerular structure, malfunction of podocytes, and disruption of the filtration barrier, leading to sclerosis and drop out of the remaining nephrons [11]. The renin-angiotensin-aldosterone system (RAAS) is thought to be overactive, which contributes also to renal hypertrophy and sclerosis. Thus, CKD is a progressive systemic disease that irreversibly damages the renal structure and alters renal function.

The disease progression of CKD affects other organ systems like cardiovascular, bone metabolism, erythropoietic hemostasis, etc. These conditions have led to intense research in both animals and humans to identify risk factors and early diagnostic markers of CKD [11]. Recent studies have implicated that Klotho, which was described as an anti-aging gene, has an association with the development and progression of CKD. The proximal tubules and distal renal tubular cells express the Klotho protein on their cell surface. α-Klotho encoded by the Klotho gene functions as a type I single-pass transmembrane glycoprotein. Two domains are present in this glycoprotein, one being a short intracellular domain of 10 amino acids and the other being an extracellular (EC) domain. There are also two 450 amino acid length internal repeats (KL1 and KL2) belonging to a family of 1 β-glycosidases, which are members of the glycosidase family of enzymes that catalyze the breakdown of complex carbohydrates. The difference between α-Klotho from family I β-glycosidases is the nonpresence of some conserved glutamic acid residues in its KL1 and KL2 regions, which regulates the catalytic activity of this enzyme family [12]. α-Klotho has three primary isoforms; a transmembrane form (mKl), a circulating soluble form (soluble Klotho (sKl)), and KL1, which is a secreted, truncated form of the protein. The main functionally active form is the soluble Klotho and is detected in the blood, urine, and cerebrospinal fluid, having paracrine effects like a hormone [13].

α-Klotho mainly functions in the distal tubular cells of the kidney, acting as an obligate coreceptor for fibroblast growth factor-23 (FGF23). FGF23 is a bone-derived hormone belonging to the FGF family that suppresses the distal tubular phosphate reabsorption and vitamin D hormone synthesis in the kidney [14]. FGF23 markedly increases in patients with CKD. FGF23 is the key regulator of phosphate metabolism, and high FGF23 levels are associated with increased cardiovascular risk [15]. Soluble α-Klotho arises from the shedding of membrane α-Klotho in the kidney by membrane-anchored proteases. In addition, Klotho is involved in the regulation of phosphorus excretion in the kidney and mineral homeostasis by regulating 1α-hydroxylase activity and parathyroid hormone (PTH) and FGF23 secretion. Serum FGF23 in many studies is considered an independent predictor for poor outcomes in CKD, as increased serum levels of FGF23 are associated with rapid progression to ESRD in CKD patients by an increased incidence of cardiovascular morbidity and mortality [16-17].

In recent years, mechanisms for the loss of membrane-bound Klotho decreasing the pathway of FGF23-stimulated signal transduction through FGFR-Klotho complexes have been studied [18-19]. The soluble Klotho controls body mineral balance by phosphorus excretion in the kidney and participates by regulating 1α-hydroxylase activity and PTH and FGF23 secretion [20]. So, in CKD patients, Klotho deficiency hampers FGF23 production, and the hyperphosphatemia resulting from it is one of the long-term consequences of CKD as well as vitamin D deficiency. Transient receptor potential cation channel subfamily V member 5 is a calcium channel protein that in humans is encoded by the TRPV5 gene. Klotho also stabilizes TRPV5 in the membrane by hydrolyzing the sugar residues of the glycan chains on TRPV5 [21]. FGF23 binds to the FGFR-Klotho complexes thereby upregulating TRPV5 protein, which further increases renal calcium reabsorption in renal distal tubules [22]. FGF23 also directly regulates sodium reabsorption in distal renal tubules by the activation of ERK1/2, SGK1, andWNK4 signal cascades, a signaling mechanism involving the FGFR-Klotho complexes [23]. Therefore, the estimation of serum Klotho protein due to calcium and sodium dysregulation in renal diseases and the novel link between FGF23 and the metabolism of these ions may have major pathophysiological implications in CKD [24].

In this study, we observed that the serum Klotho level was significantly higher in CKD patients as compared to healthy volunteers, which is contrary to various studies like Akimoto et al., 2012; Asai et al., 2012; Hu et al., 2010; Xiao et al., 2004; and Yamazaki et al., 2010, where a decrease in serum Klotho was observed with progression in CKD. Different levels of serum Klotho have been reported in many studies and can be explained by the serum Klotho assay-related variation; however, there is less data showing an increase in serum Klotho in CKD [25]. Devaraj et al., while studying CKD patients before the dialysis procedure, also showed an elevated level of soluble α-Klotho levels when compared with the control group [26]. Shimamura et al. have also concluded in their study that serum Klotho protein is increased in stage 5 CKD compared with healthy individuals [27]. The increase in serum Klotho in our study can be explained by the presence of inflammation and oxidative stress. CKD is a multifactorial disease and is closely associated with chronic inflammatory cytokines. Studies by Moreno et al., 2011; Izquierdo et al., 2012; and Hu et al., 2010, suggest that TNF-related weak inducer of apoptosis (TWEAK), along with the NF-KB pathway, can maintain serum Klotho levels, preserve renal function, and prevent renal fibrosis [5,28-29]. In some studies in mouse models like in Klotho-mutated mice, it has been documented that there is exogenous addition of soluble Klotho because of the extra renal origins of Klotho protein from the vascular endothelium, choroid plexus, or parathyroid gland.

Some studies show the overexpression of membranous Klotho protein in the tissue cultures, which suppresses the nuclear factor kappa B (NF-kB) activation, further decreasing the production of mediated inflammatory cytokines by the NF-kB mediated pathways. The mechanism involves the phosphorylation of certain serine residues in the activation domain of RelA, which is a component of the NF kB family of transcription factors [30-31]. Klotho has been seen in excess to inhibit the NF-kB pathway, leading to the decreased production of TNF-α, interleukin 6 (IL-6), and IL-12, and to attenuate the cyclosporine A-induced nephropathy in vivo and in vitro [32]. In addition, one more vital role of Klotho is to suppress NADPH oxidase 2 (Nox2) protein expression in rat aortic smooth muscle cells and decrease oxidative stress, and it can also affect retinoic acid-inducible gene I (RIG-I) mediated inflammation [33-34]. Thus, Klotho accentuation in CKD may function to ameliorate the rise in inflammatory mediators in the kidney cells, which can be considered to be a counter-regulatory mechanism.

We also studied oxidative stress in both groups in this study. The main reactive oxygen species (ROS) leading to oxidative stress are superoxides (•O2). The main source for the generation of such radicals is nicotinamide adenine dinucleotide phosphate (NADPH) oxidase in phagocytes and endothelial cells [35]. Increased activity of the RAAS leads to angiotensin II production, which activates the pro-inflammatory NF-kB signaling pathway, leading to enhanced cytokine production and generation of reactive oxygen species (ROS) [35]. Oxidative stress can further contribute to renal function decline via inflammation, hypertension, glomerular filtration barrier damage, and fibrosis. Some studies show the upregulation of the nicotinamide adenine dinucleotide phosphate (NADPH) oxidase system and downregulation of superoxide dismutase (SOD) in CKD.

The total oxidant load estimated by the FOX2 method was significantly higher in CKD as compared to control, whereas the serum total antioxidant capacity done by the FRAP assay was significantly decreased in cases (p < 0.01) when compared to the control group. These findings were in accordance with other studies [6], where the Klotho protein may function to offer resistance to oxidative stress at the cellular level by inhibiting the insulin/IGF-1 signaling pathway. This pathway negatively regulates the forkhead transcription factors (FOXOs) involved in upregulating target genes of cell cycle regulation, resistance to stress and cell longevity, and increased manganese superoxide dismutase [36]. This is one of the adaptive phenomena to remove the reactive oxygen species and provide a substantial oxidative stress resistance. The increased catalase and SOD expression facilitates ROS removal and reduces oxidative stress. Thus, Klotho-induced inhibition of insulin/IGF-1 signaling is associated with increased resistance to oxidative stress.

In our study, we found that with a decrease in eGFR, serum Klotho level increases in CKD patients by a negative correlation between them. So, we suggest Klotho can be an independent predictive biomarker for declining renal function in CKD patients.

Limitations of the study

The limitations of our study are the small sample size and the staging of CKD patients not being evaluated, so a further study should be done with larger sample size and by comparing all the study parameters of CKD patients based on the staging of the disease to look for the possibility of serum α-Klotho as a potential marker for CKD in the near future.

## Conclusions

Our study was undertaken with the objective to evaluate the role of serum α-Klotho protein in chronic kidney disease patients.

The serum α-Klotho level was significantly higher in CKD patients than in healthy volunteers. Oxidative stress was more in CKD patients but its negative correlation with Klotho protein shows the antioxidative, anti-inflammatory protective function of Klotho in CKD. As the kidney function declines as suggested by eGFR, serum α-Klotho was found to increase, which could be attributed to a decreased excretion of Klotho protein in the urine. The novelty of our study was some contrasting findings of increased Klotho protein in CKD. The correlation of serum α-Klotho protein with glomerular and tubular kidney injury biomarkers can throw better light on this ever-debatable topic of the importance of Klotho protein as a predictive biomarker for CKD patients.
